# Endometrial carcinoma in a patient with post-stroke sequelae: a case report

**DOI:** 10.3389/fmed.2026.1733931

**Published:** 2026-03-03

**Authors:** Shuangshuang Dong, Lin Dong, Lili Yang

**Affiliations:** Department of Nursing, The Fourth Affiliated Hospital, Zhejiang University School of Medicine, Yiwu, Zhejiang, China

**Keywords:** case report, endometrial carcinoma, nursing care, stroke, tracheotomy

## Abstract

**Introduction:**

Endometrial cancer is a common gynecologic malignancy, and surgical resection remains the mainstay of treatment. Perioperative complications such as malnutrition and venous thrombosis highlight the importance of comprehensive nursing care. Stroke sequelae, including hemiplegia, dysphagia, and impaired language function, further complicate management, especially when occurring simultaneously. However, reports addressing perioperative care in such patients are scarce. This study summarizes the perioperative nursing experience of a patient with endometrial cancer complicated by post-stroke hemiplegia, dysphagia, and impaired language deficits.

**Case presentation:**

A 62-year-old postmenopausal female (gravida 2, para 2) with a history of sterilization and a 7-year hemorrhagic stroke (with sequelae of hemiplegia, dysphagia, and impaired language function) presented with 3-month vaginal bleeding. Following admission, she underwent an endometrial segmented diagnostic curettage. Postoperative pathology revealed endometrioid adenocarcinoma, International Federation of Gynecology and Obstetrics (FIGO) Stage I. The patient subsequently underwent laparoscopic hysterectomy, bilateral salpingo-oophorectomy, and sentinel lymph node mapping. Postoperatively, she was transferred to the intensive care unit (ICU) for mechanical ventilation, antimicrobial therapy, and supportive care. On postoperative day (POD) 2, extubation failed, and reintubation was performed. Given the high extubation risk due to her prior tracheostomy history, a tracheostomy was conducted on POD 4. During ICU stay, enteral nutrition was administered via NG tube and discontinued due to intolerance. POD 8, she was weaned off mechanical ventilation and transferred back to the ward.

**Results:**

Proactive interventions via multidisciplinary team (MDT) discussions and nursing consultations ensured postoperative airway patency for maintaining oxygenation. Key focus areas included emphasizing the importance of nutrition, addressing the patient’s needs, protecting the patient’s skin, and preventing pressure injuries. POD 17, the patient was discharged home with a tracheostomy tube, which was removed 1 week post-discharge. The patient was able to tolerate pureed food.

**Conclusion:**

This case highlights that for patients with endometrial cancer and post-stroke sequelae undergoing Category IV procedures (laparoscopic hysterectomy, bilateral salpingo-oophorectomy, and sentinel lymph node mapping), MDT collaboration and specialist nursing consultations are critical to addressing complex perioperative care needs (airway, nutrition, skin) and achieving favorable outcomes.

## 1 Introduction

Stroke has become the leading cause of disability and mortality among Chinese adults (1). Notably, post-stroke complications are highly prevalent and impose substantial burdens on patients’ recovery. Over 70% of patients may develop motor dysfunction following a stroke (2), which severely impairs their mobility. Furthermore, 25–55% of post-stroke patients suffer from articulation and voice disorders, resulting from articulatory muscle dysfunction and impaired motor coordination for speech, these communication barriers hinder effective interaction with caregivers (3), the prevalence of dysphagia after stroke ranges from 37–78% (4), and this condition significantly increases the risk of aspiration pneumonia, a life-threatening complication that complicates post-stroke care (5), up to 62% of post-stroke patients develop malnutrition due to dysphagia (6). Collectively, these high rates of motor, speech, and swallowing impairments highlight the complexity of managing post-stroke patients, particularly when they present with additional comorbidities such as cancer.

Studies have shown that (7), the incidence of cancer among ischemic stroke patients is 9.1%, and the incidence of cancer among hemorrhagic stroke patients is 6.5%. Endometrial cancer is the most common gynecological cancer in high-income countries, with its incidence rising globally (8). The incidence of endometrial cancer in the stroke population has not been reported in current studies. Surgery for endometrial cancer is classified as a major abdominal procedure, and perioperative management, including airway care, nutrition support, thromboprophylaxis, and health education, is crucial for promoting rapid patient recovery. However, perioperative care becomes particularly challenging when the patient has post-stroke sequelae, such as language impairment, dysphagia, and motor dysfunction. To date, relevant reports on this specific population (post-stroke patients undergoing endometrial cancer surgery) remain scarce. This report, following the CARE guideline, summarizes the perioperative nursing experience of one patient with post-stroke sequelae who underwent surgical treatment for endometrial cancer.

## 2 Case presentation

A 62-year-old female with a history of bilateral tubal ligation for over 30 years and hypertension for 10 years was admitted due to postmenopausal bleeding. She had previously received medical treatment for a hemorrhagic stroke, which left her with sequelae including left eye blindness, right ear hearing loss, decreased sensation on the right side of her body, right-sided facial paralysis, and dysphagia.

Three months prior to admission, she developed unexplained mild vaginal bleeding (bright red blood with small clots) without accompanying symptoms such as abdominal pain, distension, fever, or chills. Transvaginal ultrasound showed endometrial thickening with heterogeneous echogenicity, physiological uterine atrophy, and fluid in the cervical canal with flocculent echoes (suspected clots). She refused surgical intervention at that time and was discharged; however, intermittent mild vaginal bleeding persisted. In June 2024, she presented to the Department of Gynecology at the Fourth Affiliated Hospital of Zhejiang University School of Medicine for further evaluation and management, where a preliminary diagnosis of postmenopausal bleeding was made.

On admission, her vital signs were as follows: temperature 37.2 °C, blood pressure 132/72 mmHg. Bilateral pupils were 3 mm in diameter with brisk light reflexes. She could open her eyes voluntarily and follow commands but had impaired language function, precluding normal communication; her Glasgow Coma Scale (GCS) score was 4 + D + 6 (eye opening: 4; verbal response: dysphasic; motor response: 6). Sensation was absent on the right side of her body. Muscle strength of all limbs was Grade 5, but muscle tone was increased, resulting in severe limitation of motor function. She could turn in bed independently but could not sit up or stand. Her skin condition was good (Braden Scale score: 15), and her Activity of Daily Living Scale (ADL) score was 10. A water swallowing test after admission indicated moderate-to-severe dysphagia. Nutritional risk screening was conducted upon admission, using the NRS-2002 scoring scale with a score of 2, indicating low risk. Serum albumin level was 44 g/L. Relevant examinations were completed after admission.

On June 25, she underwent partial cervical curettage under local infiltration anesthesia to manage the endometrial lesion. Postoperative pathology confirmed endometrioid adenocarcinoma (FIGO Stage I). Given her severe post-stroke sequelae, including limited mobility, prolonged bed rest, dysphagia, increased anesthetic risk during airway management, and potential postoperative rehabilitation challenges, the medical team assessed a high risk of perioperative/postoperative complications and possible stroke recurrence and venous thromboembolism (VTE). MDT involving specialists from anesthesiology, neurology, neurosurgery, nutrition, cardiothoracic surgery, and senior nursing staff was convened (June 28, 2025) for a complex case discussion, focusing on preoperative preparation, patient education, and postoperative care. On the day of the multidisciplinary discussion of difficult cases before surgery, neurosurgical experts conducted an on-site evaluation of the WST drinking water test: 30 mL of water was consumed in five separate sessions, accompanied by coughing.

The decisions were based on the experts’ experience and clinical judgment and available guidelines, including the “Prevention of Venous Thromboembolism in Gynecologic Surgery” from the ABOG (9), and the “American Society of Hematology 2019 guidelines for management of venous thromboembolism: prevention of venous thromboembolism in surgical hospitalized patients” (10). Following the MDT discussion, it was decided to perform a laparoscopic hysterectomy, bilateral salpingo-oophorectomy, and sentinel lymph node mapping on July 1 for the management of the endometrial carcinoma. Due to her dysphagia (high aspiration risk) and poor compliance, oral laxatives alone were insufficient for preoperative bowel preparation. Therefore, preoperative cleansing enemas were administered, and intestinal probiotics were co-administered to reduce the risk of inadequate bowel preparation. Due to the high risk of aspiration, a gastric tube was placed before the surgery for airway management. Due to the high risk of recurrent cerebral infarction and VTE in patients with a history of stroke, low-molecular-weight heparin anticoagulation therapy was started before surgery.

On July 1, the patient underwent the scheduled surgery. Postoperatively, she was transferred to the ICU for anti-inflammatory therapy, anticoagulation, and expectorant treatment. After the surgery, fasting was avoided, and enteral nutrition was given through nasal feeding once intestinal function was restored. Once the patient’s condition stabilized, the medication was gradually switched to oral administration. After excluding lower limb thrombosis after surgery, physical preventive measures were added based on anticoagulation, including continuous use of bilateral lower limb pneumatic compression devices for 18 h daily. POD 2, severe airway obstruction and respiratory failure occurred during extubation, requiring emergency reintubation and initiation of mechanical ventilation to ensure proper oxygenation. Intravenous methylprednisolone (40 mg daily) was given for anti-inflammatory treatment, with close monitoring of blood oxygen levels. Bronchoscopy revealed severe epiglottic edema, which obstructed glottic ventilation. To prevent recurrent respiratory issues after extubation, a tracheostomy was performed on POD 3 using a size 7 silicone tube. After blood oxygen levels stabilized, respiratory training was initiated, and she was successfully weaned off mechanical ventilation, transitioning to oxygen supplementation via a Venturi mask. The postoperative NRS-2002 score was 6 points, and enteral nutrition was started after consultation with nutrition experts.

She was transferred to a general ward on POD 8, with an ADL score of 10 and a Braden Scale score of 15. On that same day, the head nurse of the general ward invited a neurosurgical specialty nurse to conduct a nursing consultation, with the aim of providing guidance to the gynecology ward nurses on caring for tracheostomy patients.

POD 7, she developed uncontrollable diarrhea (8–10 loose stools per day). Talcum powder was applied to keep the perianal skin dry and prevent incontinence-associated dermatitis. The nutrition department diagnosed enteral feeding intolerance (serum albumin: 33 g/L). Nasogastric enteral nutrition was discontinued and replaced with parenteral nutrition. After diarrhea improved, nasogastric administration of rice gruel and liquid diets gradually resumed. Oral feeding was attempted on POD 12, and the nasogastric tube was removed on POD 14.

**Figure 1:**
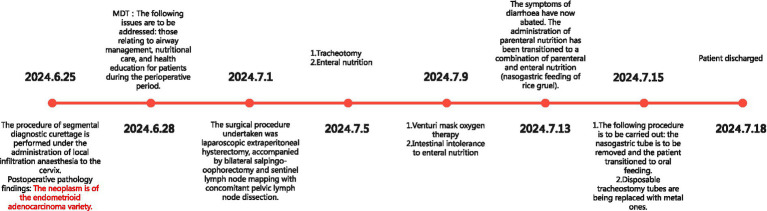
Postoperative recovery timeline for patient.

POD 14, her tracheostomy tube was replaced with an 8-gauge metal tube, and oxygen therapy was continued. She was discharged on POD 17, with stable blood oxygen levels maintained via an oxygen pillow. Her ADL score remained 10 and Braden Scale score 15 at discharge; the metal tracheostomy tube was retained and successfully removed 1 week later. By September, the patient had achieved stable recovery: she could consume pureed foods and engage in outdoor activities (e.g., sunbathing) while sitting in a specially adapted wheelchair. The patient’s diet and activity were basically the same as before the surgery. The postoperative recovery timeline is shown in Figure 1.

## 3 Discussion

This report focuses on the nursing care provided to a post-stroke patient undergoing surgery for endometrial cancer. The key nursing interventions implemented included airway management, nutritional management, skin care, prevention of VTE, health education, and follow-up care. For stroke survivors requiring surgery, a multidisciplinary approach is crucial for developing personalized nursing care plans.

The patient underwent endometrial diagnostic curettage, and the pathological result showed stage IA endometrioid adenocarcinoma. Despite an ischemic stroke in 2017, the patient’s physical function was good, and there was a chance for the early cancer to be cured by surgery. After the MDT discussion, it was considered that the patient could tolerate surgery, and the patient’s family also had a strong desire for surgery. Therefore, surgery was scheduled. Considering that the patient had post-stroke sequelae such as hemiplegic swallowing difficulties, increased surgical risks, and increased difficulty in tracheal extubation, it was necessary to plan in advance to transfer the patient to the ICU for excessive reassessment of extubation timing. The first extubation attempt in the ICU failed; bronchoscopy showed edema of the epiglottis. Considering the high possibility of recurrence after repeat extubation, tracheotomy was performed.

The case reported here highlights the importance of conducting MDT discussions on difficult cases before the surgery, with the participation of head nurses from various specialties. Coaching of nurses to master the nursing techniques not often encountered in their routine practice (e.g., respiratory tubes) is another important point taught by this case. The psychosocial and communication dimensions are important facets of nursing, reflected by the inclusion of family members in the nursing strategy to improve communication with a patient with multiple coexisting diseases. This case reflects the value of nursing consultation.

### 3.1 Airway management

For airway and tracheostomy management, the care plan (including daily assessment of readiness to wean, strict suctioning indications, ventilator maintenance, and structured oral care to reduce VAP) aligned with current evidence syntheses and best-practice recommendations for mechanically ventilated and tracheostomized patients in the ICU, which emphasize standardized assessment, secretion management, and oral hygiene bundles to prevent respiratory complications and facilitate safe liberation from the ventilator. The tracheostomy skin and stoma care measures (daily inspection, use of foam dressings to reduce device-related pressure injury) were consistent with contemporary evidence summaries on tracheostomy care and pressure-injury prevention (11, 12).

During the patient’s ICU stay, mechanical ventilation was initiated. Proper airway management during this period was crucial for ensuring the patient’s safety, preventing ventilator-associated complications, and facilitating recovery (13). The necessity of mechanical ventilation should be assessed daily. Adjustments to ventilator modes and parameters must be made precisely based on changes in these settings and the patient’s specific clinical status, with a focus on optimizing oxygen delivery and reducing oxygen consumption to restore respiratory function effectively (1). Suctioning must be performed in strict compliance with standard protocols and procedures, with rigorous assessment of suctioning indications. Suctioning should be initiated when the patient exhibits any of the following clinical indicators: (1) Significant rhonchi or wheezing; abnormal ventilator waveforms or parameter changes; (2) Dyspnea or airway obstruction; (3) Decreased oxygen saturation or deteriorating arterial blood gas results; (4)Visible excessive secretions in the oral cavity or airway; (5) Inability to produce an effective spontaneous cough; (6) Onset of acute respiratory distress; (7) Suspected aspiration of gastric or upper airway secretions; (8) Excessive sputum volume with thick consistency, accompanied by increased respiratory rate and cyanosis. Strict adherence to these suctioning indicators is crucial to prevent airway trauma from repeated suctioning and reduce the risk of infection. If airway dryness occurs post-suctioning, nebulized therapy should be administered to liquefy sputum and alleviate airway dryness.

Ventilator maintenance should be performed as follows: the heat and moisture exchanger should be replaced every 7 days or immediately if contaminated; condensate in the ventilator circuit should be removed promptly, and the condensate collection cup must be kept at the lowest point of the circuit system. Oral colonizing bacteria, dental plaque, and poor oral hygiene are closely associated with the development of ventilator-associated pneumonia (VAP). Therefore, oral care should be conducted twice daily: teeth are brushed with Xipayi Gengyin Liquid, followed by rinsing with warm water, to reduce the incidence of VAP (14).

The patient was transferred from the ICU to a general ward with a tracheostomy tube in place. The core goals of tracheostomy care are to maintain airway patency and prevent related complications, including infection, tube obstruction, and medical device-related pressure injuries.

The skin around the tracheostomy incision should be disinfected daily with normal saline or povidone-iodine, followed by replacement of the sterile gauze dressing. During this process, the incision site should be assessed for signs of redness, swelling, exudate, or bleeding. To prevent pressure injuries on the anterior neck skin, foam dressings are recommended instead of traditional tracheostomy gauze.

### 3.2 Nutrition care

The structured nutritional assessment using NRS-2002 and the combination of enteral and parenteral nutrition were planned according to international perioperative nutrition guidelines that recommend early risk screening and energy/protein targets of approximately 25–30 kcal/kg/day and 1.2–2.0 g/kg/day of protein in surgical and critically ill patients (12).

#### 3.2.1 Nutritional assessment

Nutritional support is of particular importance in perioperative management. The provision of nutritional support has been demonstrated to reduce in the early stages of infection, the incidence of complications, the duration of hospitalization, and mortality rates (15). Early nutritional assessment facilitates the timely identification of individuals at high risk of malnutrition, thus allowing for prompt and effective nutritional interventions (16). In order to reduce the patient’s recovery time, the Nutritional Risk Screening (NRS 2002) scale was employed to conduct a dynamic nutritional assessment of the patient. The aim of this assessment was to accelerate the patient’s recovery (17). The patient’s preoperative weight was recorded as 65 kilograms; she scored two on the Nutritional Risk Screening Tool. Postoperatively, she had a tracheostomy, dysphagia, and an indwelling nasogastric tube; additionally, she developed enteral nutrition intolerance. Her score on the Nutritional Risk Screening Tool increased to five, indicating a high nutritional risk. Postoperatively, her albumin level dropped to a minimum of 30.6 g/L, and a review after discharge showed an albumin level of 44 g/L.

#### 3.2.2 Nutritional pathway selection

Given the patient’s tracheostomy and swallowing dysfunction, a nasogastric tube was inserted for enteral tube feeding to prevent aspiration and choking. However, she developed enteral tube feeding intolerance during her ICU stay. After consultation with the Nutrition Department, enteral tube feeding was temporarily suspended.

Considering that enteral nutrition can improve intestinal peristalsis and digestive juice secretion, and reduce the risks of intestinal atrophy and acute stress-related gastric mucosal bleeding, the Nutrition Department recommended resuming enteral nutrition with nasogastric rice gruel once diarrhea improved. It was combined with parenteral nutrition to ensure adequate nutritional supply and maintain intestinal function (18).

#### 3.2.3 Enteral nutrition care

To prevent intraoperative aspiration, the patient underwent gastrointestinal decompression. On POD 4, enteral nutrition was initiated with Rego (a brand of enteral nutrition solution). On POD 7, the patient developed diarrhea with paste-like stools, approximately 8–10 times daily. Enteral nutrition was suspended, and the patient received 3 g of montmorillonite powder dissolved in 50 mL of warm water via tube feeding three times daily; parenteral nutrition was also increased. On POD 12, rice water was administered via nasogastric tube with gradual volume escalation, and no diarrhea occurred. On POD 14, the patient refused tube feeding by pushing the tube with hands and turning the head. A water swallowing test showed no severe choking, so the nasogastric tube was removed. Under close medical supervision, the patient was started on an oral liquid diet.

#### 3.2.4 Parenteral nutrition care

The Expert Consensus on the Safe Infusion of Parenteral Nutrition recommends that (19), the target daily intake for nutritional support is 25–30 kcal/kg/day for calories and 1.5–2.0 g/kg/day for protein. We adopted the energy requirement assessment method recommended by international guidelines, i.e., the kilogram body weight-based calculation.

For this patient (body weight: 65 kg), the required daily calories are 1,625 kcal (calculated at 25 kcal/kg/day), and the required daily protein is 97.5 g (calculated at 1.5 g/kg/day).

POD 8, the patient started receiving standardized parenteral nutrition via the central venous catheter (CVC), with the infusion pump set to 150 mL/h. Blood glucose was monitored every 6 h to maintain levels within 7.1–11.3 mmol/L. CVC patency was assessed every 12 h, and weekly maintenance was performed in strict adherence to aseptic principles to prevent catheter-related infections (CRIs) (20).

### 3.3 Skin management

Skin management strategies (Braden-based risk assessment, frequent repositioning, air mattress, moisture control, and barrier/protective products in the context of diarrhea and incontinence) reflect evidence that fecal incontinence with loose stools and high Braden risk scores are strong predictors of incontinence-associated dermatitis and pressure injury, and that structured skin-care protocols reduce IAD severity and pressure injury risk (21, 22). The tightness of the tracheostomy ties and the skin integrity of the neck and occipital area were checked twice per shift, with no pressure injuries observed. The patient had decreased sensory perception in the right limbs. On POD 7, she developed frequent diarrhea with fecal incontinence. Her Braden Scale score was 13, indicating a high risk of skin integrity impairment and incontinence-associated dermatitis (IAD). To reduce skin pressure, an air mattress was used, and the patient was repositioned every 2 h with assistance. Family members were instructed on the proper use of the bedpan and gentle wiping of stool with wet wipes. 3 M spray film and cornstarch were applied to keep the buttocks dry. No IAD or skin integrity impairment occurred until discharge (23).

### 3.4 Potential complications: prevention of VTE

VTE prevention using the Caprini risk assessment model, low-molecular-weight heparin, and mechanical prophylaxis (intermittent pneumatic compression) is supported by validation studies and guideline-based algorithms recommending pharmacologic plus mechanical prophylaxis in high-risk surgical oncology patients. These additions clarify that the nursing interventions in this case were not solely experience-based but were deliberately selected and sequenced with reference to current evidence and consensus recommendations (24, 25).

The patient was diagnosed with endometrial cancer. Studies have shown that cancer is associated with an increased risk of VTE (26, 27). The patient had a history of stroke. Her preoperative Caprini score was 6, classifying her as a high-risk patient for VTE. Since VTE can lead to recurrent stroke or even fatal pulmonary embolism, VTE prevention is particularly important.

In accordance with guidelines (10), the patient received basic prophylaxis and pharmacological prophylaxis preoperatively. Basic prophylaxis mainly included educating the patient to increase fluid intake, performing passive limb exercises, and avoiding lower limb punctures. Pharmacological prophylaxis involved subcutaneous injection of enoxaparin sodium injection (0.4 mL: 4100 AXa IU) once daily.

**Figure 2:**
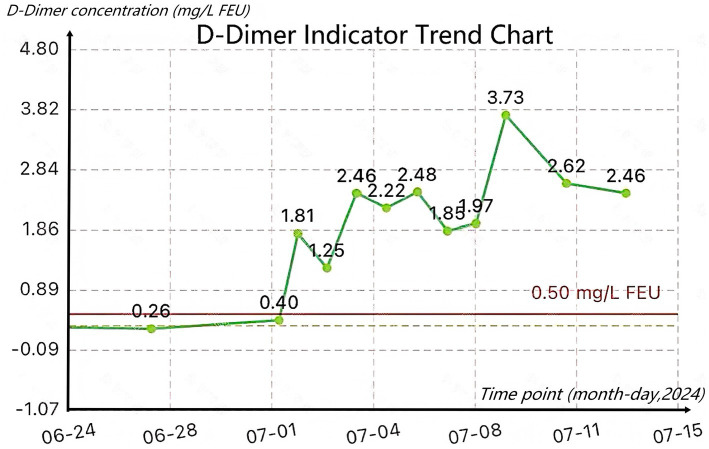
D-dimer indicator trend chart.

Postoperatively, the patient’s Caprini score for VTE was 8. After completing bilateral lower extremity color Doppler ultrasound (with no abnormal findings), physical prophylaxis was added to the preoperative prevention regimen: bilateral lower extremity pneumatic compression devices were used continuously for 18 h daily. During hospitalization, the patient’s D-dimer (DDI) levels were continuously monitored (Figure 2). No venous thromboembolic complications occurred until discharge.

### 3.5 Health education

For the patient with communication barriers, healthcare providers should allow her to use various communication symbols, such as vocalizations, gestures, postures, and intonation, during health education to ensure effective communication (28, 29). Providing a communication-conducive environment, maintaining a positive attitude, and showing sufficient patience will have a positive impact on patient communication (30).

The charge nurse of each shift conducts health education using short sentences and repetitive explanations, encourages the patient to express herself, distinguishes the tone of her responses, and observes her cooperation level. These actions help assess whether the patient understands the educational content. However, healthcare providers cannot accurately and timely assess all the patient’s needs, while her primary caregiver has a better understanding of these needs. Therefore, providing health education to the primary caregiver is of great significance for the patient’s rapid postoperative recovery.

We educated the primary caregiver on the importance of tube safety and skin protection, addressed doubts about the treatment process, and guided the caregiver to assist nurses in assessing the patient’s needs. This not only helped the patient understand and cooperate with relevant medical procedures but also kept her in a good mood and actively engaged in treatment.

Prior to the patient’s discharge, health education was provided to the primary caregiver, including guidance on feeding the patient slowly to prevent choking; continuing limb rehabilitation exercises to reduce muscle atrophy; avoiding sexual activity and tub baths for 3 months; and repositioning the patient multiple times daily, plus performing back percussion to promote sputum excretion.

At discharge, the primary caregiver was assessed and confirmed competent in tracheostomy tube care, blood pressure monitoring, and oxygen saturation monitoring.

### 3.6 Psychological considerations

The family members may feel sad and upset, but considering that the patient’s physical condition was relatively good at the time and the cancer was in its early stages, the family members still need to have an active attitude towards treatment. Our nursing team attaches great importance to the patient, with senior nurses providing dedicated care, communicating with the patient and family members every day, guiding their recovery, and the family members cooperating well. In the case reported here, the patient was dysphasic due to her stroke. Since her family members were used to that limitation and had managed over time to establish communication with the patient, they were included in the care plan to ensure more effective communication between the patient and the healthcare professionals.

However, there are certain limitations in our care. A review of the nursing process in this case revealed that psychological care for the patient may have been insufficient. Therefore, in future clinical practice, healthcare providers should strengthen psychological assessment and intervention for such patients. In addition, no standardized neurological assessment was performed. Such an assessment could help nursing management and should be performed in similar cases.

### 3.7 Patient follow-up

The patient was hospitalized for 27 days and underwent follow-up 1 week after discharge. At this time, the tracheostomy tube had been removed, the patient could eat pureed food, her general condition was good, and there was no vaginal bleeding or abdominal pain.

At 3 months after discharge, the patient failed to attend the scheduled hospital review. A telephone follow-up was conducted, during which the primary caregiver stated that the patient was in good condition and could use a special wheelchair to go to the park for sun exposure.

In summary, the complex perioperative care needs of patients with endometrial cancer and post-stroke sequelae call for more effective response strategies. Specifically, conducting a comprehensive preoperative evaluation for such patients (including assessment of stroke-related motor function and cancer surgical tolerance) is of great importance. Furthermore, the MDTs can ensure the provision of high-quality diagnostic and therapeutic recommendations as well as optimal treatment plans for patients with multiple comorbidities during the perioperative period. We believe that in the future, nurses can serve as initiators of MDTs, collaborating with specialized nurses and nursing experts to deliver more comprehensive nursing care to patients with multiple comorbidities.

Notably, when highly specialized nursing issues arise in ward nursing (such as the complex perioperative care of the aforementioned patients), specialist nursing consultations also play a crucial role: they not only help resolve clinical nursing challenges but also facilitate the timely identification of problems and proactive intervention, this positive role is key to preventing the occurrence of severe complications, and further complements the collaborative support of MDTs in improving the quality of care for patients with multiple comorbidities.

### 3.8 Major decision points

First, in view of the patient’s severe post-stroke dysphagia and high aspiration risk, a nasogastric tube was placed preoperatively to secure an enteral feeding route and facilitate safer airway and nutritional management around the time of surgery. Enteral nutrition is recommended for stroke patients who cannot safely swallow, both to prevent malnutrition and to reduce complications such as aspiration pneumonia and poor functional recovery. Early initiation of enteral tube feeding (within the first few days after recognizing dysphagia or nutritional risk) is associated with improved functional outcomes and lower complication rates in stroke populations, and nasogastric feeding is widely regarded as an appropriate early route in patients with severe dysphagia. Dysphagia after stroke is highly prevalent and is strongly linked to malnutrition, dehydration, aspiration pneumonia, and worse post-stroke prognosis, supporting proactive decisions about early tube placement rather than prolonged fasting or delayed nutritional support. In the postoperative period, once intestinal function returned, enteral nutrition via the nasogastric tube was resumed promptly to avoid extended fasting, consistent with guidance that early enteral nutrition can improve neurological and overall outcomes in stroke patients. As the patient’s condition stabilized and swallowing function permitted, medications and diet were gradually transitioned from tube to oral administration, in line with evidence that recovery of safe oral intake is associated with better long-term outcomes and that tube feeding should be withdrawn as soon as adequate oral intake is feasible (31–33).

Second, considering the patient’s history of stroke and malignancy, she was classified as at high risk for perioperative venous thromboembolism, including recurrent cerebrovascular events and deep vein thrombosis or pulmonary embolism. Current gynecologic surgery and gynecologic oncology guidelines recommend systematic VTE risk assessment (e.g., Caprini) and dual prophylaxis (pharmacologic plus mechanical) for high-risk patients undergoing cancer surgery, provided that bleeding risk is acceptable. Perioperative protocols in gynecologic oncology have shown that expanded thromboprophylaxis regimens, including preoperative administration of unfractionated or low-molecular-weight heparin at the time of anesthesia induction and extended postoperative LMWH in women with malignancy, significantly reduce symptomatic VTE without increasing bleeding or infectious complications. More broadly, perioperative anticoagulant prophylaxis initiated shortly before surgery (roughly within 2 h before to 4 h after incision) is more effective at preventing postoperative VTE than regimens that omit this period, and physical measures such as intermittent pneumatic compression are an important adjunct, particularly in high-risk surgical or neurologic patients. In this case, prophylactic low-molecular-weight heparin was started in the preoperative period to address the elevated risk of recurrent thromboembolic events, and, after lower-limb deep vein thrombosis was excluded postoperatively, pharmacologic prophylaxis was complemented by continuous bilateral lower-limb intermittent pneumatic compression for most of the day. This combined pharmacologic–mechanical strategy is consistent with contemporary recommendations for high-risk gynecologic oncology patients and with evidence that appropriately prescribed VTE prophylaxis can reduce deep vein thrombosis and pulmonary embolism by more than half in hospitalized and surgical cohorts (34, 35).

## Data Availability

The original contributions presented in the study are included in the article/supplementary material, further inquiries can be directed to the corresponding author.
